# What matters to you? Improving the adoption of shared decision-making for birth planning in women with chronic hypertension: a multicentre multiple methods study

**DOI:** 10.1136/bmjopen-2024-094607

**Published:** 2025-06-17

**Authors:** Rebecca Whybrow, Lucy Chappell, Louise Webster, Joanna Girling, Heather Brown, Hannah Wilson, Marcus Green, Jane Sandall

**Affiliations:** 1 Florence Nightingale Faculty of Nursing, Midwifery and Palliative Care, King’s College London, London, UK; 2 Women’s Health Academic Centre, King’s College London, London, UK; 3 Obstetrics and Gynaecology, Chelsea and Westminster Healthcare NHS Trust, London, UK; 4 Chelsea and Westminster Hospital NHS Foundation Trust, London, UK; 5 Brighton and Sussex University Hospitals NHS Trust, Brighton, UK; 6 Royal College of Obstetricians and Gynaecologists, London, UK; 7 Action on Pre-Eclampsia, Evesham, UK

**Keywords:** OBSTETRICS, Maternal medicine, Hypertension, Decision Making, Pregnancy, Pregnant Women

## Abstract

**Abstract:**

**Objective:**

To explore the role of shared decision-making (SDM) in the implementation of evidence-based practice in women with chronic hypertension planning birth and investigate the barriers and the facilitators in the provision of antenatal care.

**Methods:**

A multimethod multisite approach was used including case-note review (n=55) and structured observations (n=18) to assess the provision of third trimester antenatal care. The barriers and facilitators to implementation were identified from semistructured qualitative interviews with healthcare professionals (n=13) and pregnant women (n=14) using inductive thematic analysis. The findings were integrated and evaluated using the ‘Three Talk Model of Shared Decision-making’.

**Setting and participants:**

Pregnant women with chronic hypertension, some with superimposed pre-eclampsia and their principal carers at three National Health Service hospital trusts.

**Results:**

Healthcare professionals delivering care to pregnant women with high blood pressure were aligned with most communication practices (set out in the Calgary-Cambridge communication guide). Pregnant women with hypertension who described being engaged in shared decisions about birth developed a trusting relationship with their maternity team. Despite frequent caesarean section birth (52%) and early term birth (median gestation at delivery 38 weeks (IQR1 37 weeks, IQR3 39 weeks) identified by case-note review; integrated data (observations, case-note review and qualitative interviews) found pregnant women with high blood pressure were not regularly provided with personalised information based on what they would find helpful, encouraged to share their own thoughts or offered choice in relation to timing or mode of birth. Uncertainty regarding the evidence around optimal timing of birth was the main barrier identified by professionals. Facilitators included training for professionals in SDM, midwife-led antenatal classes for high-risk women and multiprofessional clinics.

**Conclusions:**

Strategies to promote more widespread adoption of SDM are likely to improve the experiences of women with high blood pressure making decisions about childbirth.

**STRENGTHS AND LIMITATIONS OF THIS STUDY**

- About two-fifths of women who participated in this study were from black, Asian and minority ethnic groups, providing a diverse range of voices.
- Multimethodological approaches and a theoretical framework improved the reliability, validity and generalisability of the study.
- Structured observations were carried out using a validated tool with high inter-rater reliability.
- Data collection occurred in 2018; however, in 2023, the Care Quality Commission Maternity Survey reported unchanged or declining maternity experiences nationally in women with pre-existing medical conditions, indicating that our results likely remain relevant to current practice.
- The case-note review relied on electronic coding of pregnant women with chronic hypertension; however, at one hospital, it is likely, based on population trends, that there were additional women with chronic hypertension who had not been coded correctly, and as such were not identified for case-note review.

## Background

 Chronic hypertension affects around 1.5% of pregnancies, with rates rising in the UK and in other comparable countries such as the USA.^1 2^ A systematic review has previously reported that women with chronic hypertension are at substantial risk of morbidity from superimposed pre-eclampsia (25.9%), while their babies have a neonatal unit admission rate of 20.5% and perinatal death rate of 4.0%.^3^ Chronic hypertension in pregnancy disproportionately affects black and ethnic minority women in terms of prevalence and severity and is, therefore, a determinant of maternal health inequalities.^4^ Decision-making to reduce morbidity and optimise birth outcomes includes planning ‘timing of birth’ and ‘mode of birth’. This involves discussion of balancing potential risks and benefits to the woman with potential risks and benefits to the unborn baby, that also considers women’s preferences, as both timing and mode of birth are preference-sensitive decisions. Resultingly, the National Institute for Health and Care Excellence (NICE) Hypertension in Pregnancy guidelines^5^ advise women with chronic hypertension whose blood pressure is lower than 160/110 mm Hg after 37 weeks, with or without antihypertensive treatment, that timing of birth and maternal and fetal indications for birth be agreed between the woman and the senior obstetrician, given the lack of definitive evidence,^5 6^ while the NICE Caesarean Birth guidelines recommend a shared decision-making (SDM) approach is adopted when supporting women to plan their mode of birth.^7^

SDM is the principal approach to health-related decision-making in England and Wales as outlined in the NICE SDM guidelines.^8 9^ It is an approach where clinicians and individuals accessing healthcare share the best available evidence when faced with the task of making decisions, and where patients or persons are supported to consider options, to enable them to make informed decisions.^10^ Since 2008, SDM principles have underpinned GMC (General Medical Council) guidance on informed consent. Crucially, the guidance recommended that doctors use specialist knowledge and experience and clinical judgement, and the patient’s views and understanding of their condition, to identify which investigations or treatments are likely to result in overall benefit for the patient. The doctor explains the options to the patient, setting out the potential benefits, risks, burdens and side effects of each option, including the option to have no treatment.^11^ Following the 2015 Montgomery versus Lanarkshire National Health Service (NHS) Trust, the court ruled consent should further be based on what the patient may want to know, not what the doctor thinks they should be told.^12^ This approach is also informed by the growing body of evidence that SDM improves patients’ satisfaction with decision-making and can improve health outcomes (particularly in disadvantaged populations).^13^ Despite the clinical and legal imperative to adopt SDM, the extent to which women with chronic hypertension are involved in birth planning decisions has not been investigated.

## Methods

Informed by the Three Talk Model,^14^ the study aims to explore the role of SDM in the implementation of evidence-based practice in women with chronic hypertension. Objectives: (1) describe the care being implemented for women with chronic hypertension in pregnancy across England; (2) investigate the current uptake of evidence-based practice and explore the determinants associated with their implementation for women with chronic hypertension in pregnancy and (3) investigate the barriers and facilitators in the provision of antenatal care for women with chronic hypertension in pregnancy. The research was informed by the 2010 NICE hypertension in pregnancy guideline (which sets out the recommendations in terms of birth planning)^5^ and by the ‘Patient experience in adult NHS services guideline’, (which sets out recommendations for actively involving patient in decisions about their care through information provision and SDM).^8^

Birth planning was assessed through (1) a review of maternity case notes of women who had already given birth, a method that assessed the documentation of birth plans and subsequent birth outcomes in each woman’s maternity record and (2) semistructured observations of third trimester antenatal consultations to assess birth planning practices that would not normally be documented or are more difficult to capture, such as in-consultation discussions and occurrence of SDM. The evaluation of the barriers and facilitators to the implementation of birth planning was assessed through qualitative interviews (with the same women and healthcare professionals (HCPs) who participated in the observation phase). These data were collected alongside published data pertaining to the management of hypertension in pregnancy.^12^ The data presented in this paper are novel and non-duplicative, though the methodology was used for parallel objectives in the wider CHAMPION (Cronic Hypertension In Pregnancy Implementation Study) study. The study draws on The Three Talk Model as a theoretical framework to guide data collection, analysis and interpretation. The Three Talk Model sets out the characteristics required to implement SDM effectively. These include ‘team talk’ (working together, describing choices, offering support and asking about goals), ‘option talk’ (discussing alternatives using risk communication principles) and ‘decision talk’ (getting informed preferences to make preference-based decisions). These stages are underpinned by the principles of active listening and deliberation.^14^

Birth planning in women with chronic hypertension in pregnancy was assessed between November 2017 and December 2018. Inclusion criteria: Pregnant women with chronic hypertension, aged over eighteen and able to give consent. Exclusion criteria: none. The study was conducted at three NHS Trusts with typical configurations of services for pregnant women with hypertension in the UK. Hospital Trust 1 was a tertiary city centre hospital with a newly formed specialist service that included consultant obstetricians, obstetric physicians and midwives who provided antenatal and intrapartum care to women with chronic hypertension within a specialist clinic; Hospital Trust 2 was a suburban district general hospital with a consultant-led antenatal clinic with antenatal midwives alongside providing care to women with a variety of pre-existing medical conditions and Hospital Trust 3 had both a tertiary and a semirural hospital with a joint obstetric and physician-led clinic and usual community-based midwifery care. The NICE hypertension in pregnancy guidelines (2010) had been adopted into local clinical guidelines at all three participating NHS Trusts for several years prior to the assessment of implementation.^5^

### Case-note review

Timing and mode of birth, as well as documented decision-making, were assessed through review of 100 maternity case notes of women coded with chronic hypertension identified from the electronic maternity records (32, 33, 35 women per Trust). The entirety of the case notes was reviewed, and the birth plan was usually documented between 28 and 36 weeks by the consultant obstetrician. At hospital Trust 1, all women with CHT (Chronic Hypertension) who had given birth over the final 3 months of 2017 were included, whereas at hospital Trusts 2 and 3, all women coded with CHT who had given birth in 2017 were included, as these Trusts had approximately a quarter of the number of women with chronic hypertension each year. In the UK, many women have abridged electronic maternity records and extensive handheld paper notes that are carried throughout pregnancy but are stored thereafter in the hospital. Both the electronic system and paper notes were obtained in the case notes review of care. Due to the use of varying terms for hypertension on the electronic system, some women identified for case-note review were excluded as they did not have chronic hypertension when the full case notes were examined. Other reasons for exclusion included early miscarriage and transfer of care to another maternity unit. Data extraction based on items within the NICE hypertension in pregnancy guidelines (2010)^5^ was completed by two midwife researchers (RW and HW), and minor discrepancies were resolved by discussion between the two researchers. It was not necessary to include a third reviewer as no major discrepancies were identified. Unclear or absent documentation including height, weight and body mass index (BMI) or antenatal blood pressure recordings was recorded as missing data. Severe hypertension was defined as systolic blood pressure greater than or equal to 160 mm Hg systolic or diastolic blood pressure greater than or equal to 110 mm Hg.

### Observations

Women with chronic hypertension were purposively sampled at their first obstetric antenatal appointment and, based on the availability of the midwife researcher, were approached consecutively along with their respective HCPs until data saturation occurred. Staff and women gave written informed consent. Two women declined recruitment to the study. Data from earlier appointments, which focused on hypertension management, have been reported in an earlier BMJ Open paper.^15^ This paper reports on third trimester appointments when birth planning conversations usually occur. 18 third trimester antenatal appointments involving 18 women with chronic hypertension and their respective doctors (14), and midwives (4) were observed by a midwife researcher (RW) at the three NHS Trusts. The midwife researcher carried out the observations in person in the antenatal clinic room. During observations, data about birth planning were recorded using the Calgary-Cambridge communication guide^16^ chosen for validity in relation to the research question and its high inter-rater reliability. Professionals were told antenatal care was being observed but not specifically using the Calgary-Cambridge communication guide. Attainment of each section and subsections was established through the analysis of all 18 appointments using descriptive statistics.^16^

### Semistructured interviews

Views about planning birth in women with chronic hypertension were collected from nine doctors and four midwives who were providing the observed third trimester antenatal appointments. The interviews were carried out by a midwife researcher (RW) following informed consent whereby participants were provided with information about the study and given time to ask questions prior to providing consent and took place in privacy away from the clinical setting. The interviews were audio transcribed, coded and thematically analysed using a deductive approach^17^ informed by The Three Talk Model for SDM.^14^

Semistructured interviews with 14 of the 18 women recruited for antenatal observations were carried out in the third trimester with informed consent. Two women consented to have their antenatal appointment observed but declined to be interviewed, while two women were delivered after induction of labour immediately following their appointment and could not take part in the follow-up interview. Women were asked about their experiences of their third trimester antenatal care using an interview schedule which reflected the concepts from the International Consortium for Health Outcome Measure (ICHOM) maternity standards sets^18^ which include women’s overall satisfaction with their care during the third trimester of pregnancy; satisfaction with information provision and decision-making. ICHOM standards are internationally recognised measures that evaluate health outcomes that are important to patients (or pregnant women) and are used to improve local healthcare and compare outcomes internationally.^18^ The closed survey questions were turned into open-ended questions to explore in-depth the quality of antenatal care provided (online supplemental file 1). The interviews were carried out by a midwife researcher (RW) and took place away from the clinical setting, with assurance that discussions would not be shared with HCPs and that participation or non-participation would not influence their care. The interviews were audio transcribed, coded and thematically analysed using an inductive approach. Women’s experiences were analysed to improve understanding of their birth planning needs.

### Data analysis

The quantitative and qualitative data were analysed separately before being integrated. Descriptive analysis and summary statistics were used for the quantitative data. The semistructured interviews were thematically analysed by researchers (RW, JS and LC) using inductive techniques and typically lasted between 30 and 60 min.^19^ This included probing the inductively generated qualitative themes that related to implementation by the midwife researcher (RW) who collected the data, then with a second and third researcher (JS and LC) interpreting and discussing the final interpretation of integrated data. Rigour was maintained through member reflection, attention to interview and transcription quality and systematic analysis. The multiple methods data were integrated and analysed using the Three Talk Model.^14^ Rigour was improved using multiple data sources and a multiple methods integration checklist.^20^ Researchers were aware of, and sensitive to, the way in which their roles as midwives and doctors may have shaped the generation and analysis of the qualitative data.

### Patient and public involvement

A patient participant involvement (PPI) group consisting of women with experience of hypertension in pregnancy (n=7) and PPI members of the Maternity Applied Research Collaboration South London (n=15) provided feedback on the design of the study, research questions and outcome measures. The views of black, Asian and minority ethnic women were purposively sought as they are disproportionately represented in the chronic hypertension in pregnancy population. PPI focus groups discussed what aspects of care were important to evaluate. This included the information women were given during pregnancy and whether women were involved in decisions about their care. They also provided constructively critical feedback on the participant information leaflets and consent forms.

## Results

Antenatal care for women with chronic hypertension was provided by obstetricians and midwives at all three hospitals. In two of the hospitals, women with chronic hypertension had designated midwives attached to the obstetric clinic. Approximately two-fifths of those recruited to the study were of black, Asian and minority ethnic backgrounds, approximately one-third had a BMI over 30 kg/m^2^ and approximately one-third were over the age of 35. These demographics are aligned with Webster and colleagues’ UK CHT cohort study published in 2019.^21^ Hospital Trust 1 had four times the population of women with chronic hypertension compared with the other two units, including a large black minority ethnic population (table 1).

**Table 1:** Maternal demographics of women observed and interviewed

Women demographics	Observed % (n/18)	Interviewed % (n/14)	Case notes % (n/55)
Ethnicity			
Asian	11.1 (2)	7.1 (1)	14.5 (8)
Black	33.3 (6)	21.4 (3)	32.7 (18)
White other	22.2 (4)	28.6 (4)	14.5 (8)
White British	33.3 (6)	42.8 (6)	27.3 (15)
Any other	0.0 (0)	0.0 (0)	0.0 (0)
Parity at booking			
0	44.4 (8)	50.0 (7)	27.3 (15)
1	33.3 (6)	28.6 (4)	38.2 (21)
2 or more	22.2 (4)	21.4 (3)	34.5 (19)
Age at booking			
20–34	66.7 (12)	64.2 (9)	41.8 (23)
35–39	22.2 (4)	28.6 (4)	38.9 (21)
40–44	11.1 (2)	7.1 (1)	20.4 (11)
Median age (IQR1–IQR3)			35 (31.0–38.5)
BMI at booking			
<18.5	0.0 (0)	0.0 (0)	1.9 (1)
18.5–24.9	27.8 (5)	28.6 (4)	25.0 (13)
25–29.9	38.9 (7)	50.0 (7)	25.0 (13)
30–34.9	22.2 (4)	14.3 (2)	21.2 (11)
35–39.9	11.1 (2)	7.1 (1)	11.5 (6)
≥40.0	0.0 (0)	0.0 (0)	7.7 (8)
Median BMI (IQR1–IQR3)			30 (25.3–36.1)

Women interviewed are a subset of those observed.

BMI, body mass index.

### Timing of birth

The case-note review of timing of birth found the gestation at which women with chronic hypertension in pregnancy gave birth with a median gestation of 38 weeks (IQR1 37 weeks–IQR3 39 weeks). There was consistency between planned and actual timing of birth. One in six women had their birth planned for the 37th week of pregnancy, one in four in the 38th week of pregnancy and one in six had their birth planned after 40 weeks. One in 10 women had no planned date of delivery documented (table 2).

**Table 2:** Case-note review of the care provided to pregnant women with chronic hypertension at three NHS Trusts

	Planned gestation of birth % (N)	Actual gestation at birth % (N)
Gestation (weeks)		
<34+0	5.5 (3)*	5.5 (3)
34+0–36+6	14.5 (8)*	14.5 (8)
37+0–37+6	16.4 (9)	18.2 (10)
38+0–38+6	27.3 (15)	30.9 (17)
39+0–39+6	10.9 (6)	20.0 (11)
40+0–40+6	7.3 (4)	9.1 (5)
≥41+0†	9.1 (5)	1.8 (1)
No plan documented	9.1 (5)	
Median (IQR1–IQR3)	38 (37–39)	38 (37–39)
Mode of birth	Planned mode of birth % (N)	Actual mode of birth % (N)
All caesarean section birth	38.2 (21)	52.7 (29)
Planned caesarean section	38.2 (21)	38.2 (21)
Emergency caesarean section		14.5 (8)
All vaginal births	61.8 (34)	47.3 (26)
Instrumental birth		5.5 (3)
Spontaneous vaginal birth		41.8 (23)

* 11 women had their birth expedited prior to 37 weeks due to maternal/fetal compromise and, therefore, planned date of delivery is recorded as date the decision for delivery was made.

† Induction of labour planned after 41 weeks gestation if spontaneous labour has not occurred.

NHS, National Health Service.

### Mode of birth

The case-note review found almost three-fifths of women had planned for a vaginal birth (two-fifths planned for a caesarean section). Nevertheless, less than 50% of women with chronic hypertension in pregnancy gave birth vaginally compared with 70% in the general population.^22^

### Decision-making

One-third of case notes reviewed had documentation of women’s involvement in planning their birth (timing or mode of birth) (table 3). Women were more likely to be involved in birth planning if they gave birth at term; two fifths of notes had documentation of women’s involvement in planning of mode and timing of their birth if they gave birth after 37+0 weeks’ gestation, compared with just 1 in 10 women who gave birth before 37 weeks’ gestation. Explicit documentation of women’s involvement in birth planning was more common in those who had a planned caesarean section (two-fifths) compared with those who planned vaginal birth (one-fifth).

**Table 3:** Case-note review of pregnant women’s involvement in decision-making at three NHS Trusts

Woman’s involvement in birth planning documented	Case notes % (N)
Yes	34.6 (19/55)
No	65.4 (36/55)
Women’s involvement by gestation at delivery documented	
<37 weeks	9.1 (1/11)
≥37 weeks	40.9 (18/44)
Women’s involvement by planned mode of birth documented	
Planned caesarean section	42.9 (9/21)
Planned vaginal birth	29.4 (10/34)

NHS, National Health Service.

All third trimester appointments that were observed had some discussion about timing or mode of birth; two thirds of appointments observed occurred after 34 weeks and involved making decisions about birth. Structured observations of antenatal care found that overall, HCPs’ communication was aligned with the Calgary Cambridge Communication Guide^16^ (online supplemental file 2). However, SDM and items relating to patient involvement were less frequently observed. One-fifth of women were offered personalised information based on what they would find helpful, half of women were encouraged to share their own thoughts and preferences and one-third of women were offered choice in relation to deciding timing or mode of birth.

### Women’s experiences of decision-making

#### Theme 1: team talk (agenda setting and choices)

Half of women interviewed discussed the importance of setting the agenda earlier in their pregnancy than is currently normal practice. Women understood their pregnancy was complex and that birth planning was also likely to be complex, and as such wanted time to consider their options. Women’s experiences of being offered choice around birth planning were variable. Women who were offered choice were more satisfied than those who felt they needed to negotiate options or were told choice wasn’t available to them (box 1).

Box 1 **Barriers and facilitators to shared decision-making adoption experienced by women**

**Theme 1a: team talk (agenda setting)**

I’ve asked, and my response so far has been we’ll see about it, like we’ll talk to you about it closer to the time. Which isn’t very helpful for me! (laughs) I like to plan woman 11

Well, they told me from early on what, they always made me aware of what could possibly happen if I continued to have high blood pressure throughout. So, I knew from like day one what would happen woman 3

I think I initiated the conversation actually, I don’t, she probably had it in mind to talk to me about it, but I’d initiated it, because it was getting to that point in the pregnancy, we were in the third trimester, I was beginning to think about the end date, the endgame woman 4

**Theme 1b: team talk (offering choice)**

So, I was offered either to be induced at 38 weeks or, or to wait till 40 weeks. And that was my decision. From my perspective I had this like very subjective feeling that I, I should deliver this baby earlier, and that was the right call (laughs). So, yeah, so I appreciated that I had, I had a choice. Woman 5

He said when I went to my last appointment that he would have induced me at 38 weeks because of my blood pressure, but because I, I then questioned and said my blood pressure was fine, he then increased it to 39 weeks. Woman 14

What Dr X said was that NICE guideline, say that it, you can choose, but that the hospital policy is, or the commissioning thing, or something, is that they don’t do elective caesareans here. Woman 13

**Theme 2: option talk (information provision)**

I think the only thing that would be more helpful is information that’s a bit more tailored to me. So, you know, I was asking, asking kind of Dr X about the chances of ending up with an emergency C/section, I think she said something like, I can’t remember exactly what the percentage is, like 70% of induced labour leads to a vaginal delivery, but I guess for me particularly there’s kind of a few risk factors, so maybe information that’s a bit more tailored would be helpful. Woman 6

I think, sometimes the two sides (hypertension and diabetes) can talk to each other a little bit more and be slightly more joined up. Woman 10

Yeah, so I, I attended the antenatal class run by the midwife team a few weeks ago…it was really helpful actually….just kind of knowing what the process is, like managing expectations a bit. I think it’s, it’s always the, the unknown, it’s quite scary. Woman 6

**Theme 3: decision talk (balancing risks and decisional conflict)**

I asked for some more nifedipine, all of a sudden there’s panic about whether or not the baby needs to be delivered. Yeah, so it’s a trade-off, you know, and actually our daughter had respiratory distress syndrome, and ended up in NICU, I’d been induced at 37+2. But, so we’re just keen to bake this one for as long as possible, just to try and reduce the risks. And then I know it’s a trade-off between risk to me and potentially the baby of abruption, vs you know keeping her in for a few more days. Woman 10

cause the diet-controlled gestational diabetes, they said you wouldn’t need to be induced till 40+6, so really, technically I should go on to that, but, they, the consultant was kind a saying well it’s 38 weeks for blood pressure, and then it would be 39 weeks because you’re gestational [(diabetic]), so they, they were like pushing that induction further forward than it needs to be, and it’s not part of the, the NICE guidelines and stuff, it’s not what they would recommend. So, but I mean I’m, I’m kinda like in two minds whether I do it just to get the baby out earlier or take the risk of just waiting for a bit longer. So, it’s like, it’s really difficult. Woman 14

**Theme 4: active listening and deliberation**

I felt respected, and therefore I felt that I could trust them ‘cause I didn’t feel that they just hadn’t listened to me. So actually, I think that probably helped to reinforce my feeling that they were trustworthy, because they didn’t just try to railroad me into a decision that I didn’t want to take. Woman 13

I’m being induced 3 weeks earlier, so I guess if we kind of started talking about this maybe like mid, like, maybe sort of mid-way through the pregnancy rather than right at the end of the pregnancy that would be, would have given me a bit, bit more time to think about it Woman 10

I think you could do with someone else just backing you a little bit. ‘Cause the consultants are very much, they don’t wanna take the risks, so they just would rather it was done, and not worried about it, but, whereas your midwife would probably be a bit more on your side Woman 26

#### Theme 2: option talk (information provision)

Women with chronic hypertension described wanting more personalised information about timing and mode of birth. In circumstances where they had comorbidities such as diabetes, women wanted the HCPs to work collaboratively when making decisions about birth. Women described wanting information in more varied formats such as apps, videos and images. Antenatal classes run by midwives for women with complex pregnancies were a particularly valuable source of information for those who had access to them (box 1).

#### Theme 3: decision talk (balancing risks and decisional conflict)

Women’s experienced decision-making as dynamic due to the changing clinical picture. Some women were also balancing risks related to comorbidities with care being managed across different specialist pregnancy clinics. Decisional conflict was frequently described by the women in this study (box 1).

#### Theme 4: active listening and deliberation

Women described being listened to by HCPs as an important facilitator to building trust and enabling decision-making. Women reported they needed more time than they were given to consider their options (box 1).

### HCPs’ experiences

#### Theme 1: team talk (agenda setting and choices)

The paucity of research evidence into the optimal time of birth for women with chronic hypertension (without superimposed pre-eclampsia) was highlighted by the clinical team. Most of the team felt birth discussions should start in the third trimester of pregnancy once more clinical information had been obtained. However, some professionals recognised the value in earlier agenda setting (box 2).

Box 2 **Barriers and facilitators to shared decision-making adoption experienced by healthcare professionals**

**Theme 1: team talk (agenda setting and choices):**

I don’t think yet that there’s evidence to, to suggest that, you know, one size fits all and that we should be inducing everybody at 37 weeks or everyone at 40 weeks. Dr, 9

I’m not particularly fussed about having a 37 weeks or 39 weeks or any of the other one, I think if everything’s going well then I would have 40 weeks as my cut-off. Dr, 14

So, by 34 weeks we hope that we’ve had a conversation about, if all goes well this is when we’d be hoping to deliver you….I don’t think it’s massively helpful to have, right at the beginning of the pregnancy, ‘cause actually the care in the first, up to 34 weeks isn’t going to be any different. Dr 1

**Theme 2: option talk (information provision)**

it’s a mixed class so we do it with the other high-risk team, who also caseload sickle-cell ladies, but I would say, because everybody’s different, some people might be having a planned C/section, or, or it could be early, we’ve got foetal cardiac ladies who deliver early, and so we do cover all of those things, and we’ll be, you know, talking about hand-expressing if we know they’re more likely to have a prem delivery, and all of those sorts of issues. So, it’s probably a bit more realistic to them than just straightforward antenatal classes. MW, 17

I think that’s just something we do individually in the clinic, and also pros and cons of the induction, cons of induction as well, and I think that’s more done by us in clinic Dr, 13

An infographic, a bit more information, so you can talk around the thing, and then you can send it electronically to her phone or email or whatever, so that she’s got a copy of that information. And so then, it’s a way to standardise the information, and to, to prevent it, to present it in a, in a more visual way. Dr, 9

**Theme 3: decision talk (approaches to decision-making)**

I think it depends on the clinician if I’m honest. Some people are very pro sort of talking about well we could do this, we could do this, and, you know, what you want to do, and other people are sort of, you must have an induction! DR, 13

I always kind of discuss the different possibilities of options that might happen in terms of kind of timing of delivery, and in, kind of in terms of mode of delivery we discuss what the women want, and their preferences, but it’s often kind of like, well, we’re waiting on the obstetricians to make a plan. MW, 7

I suppose, you’ve got the team of, you’ve got your obstetrician, you’ve got your physician, you’ve got your patient, person, and you’ve got your midwife. And sometimes you’ll have your diabetes nurse. And then, you know, when you’re discussing things like well, when do you think you should deliver this baby, then you have a, integrated discussion between all the people there. And you throw round a few ideas, and you come up with a, an agreed solution, you see, and I think that’s a really good way of doing it. So, you know, the, I mean, do we do it at 40 weeks, do you do it at 39 weeks, do you do it at 38 weeks? Dr, 16

#### Theme 2: option talk (information provision)

A personalised approach to sharing of information occurs in antenatal appointments, but professionals report wanting conversation aids to support information sharing in this setting. Midwife-led antenatal classes for women with complex pregnancies were described as an efficient and personalised approach to information sharing.

#### Theme 3: decision talk (approaches to decision-making)

Decisions about the timing of birth were described differently by midwives and doctors. One doctor described the approach to decision-making as clinician-dependent. A collaborative multiprofessional SDM approach was described by doctors and midwives working in a multiprofessional specialist clinic.

## Discussion

HCPs delivering care to pregnant women with high blood pressure were aligned with most communication practices (set out in the Calgary-Cambridge communication guide). Pregnant women with hypertension who described being engaged in SDM about ‘timing’ and ‘mode of birth’ described developing a trusting relationship with their maternity team and described being more satisfied with their care. Despite frequent caesarean section birth (52%) and early term birth (median gestation at delivery 38 weeks (IQR1 37 weeks, IQR3 39 weeks) identified by case-note review; integrated data (observations, case-note review and qualitative interviews) found pregnant women with high blood pressure were not regularly provided with personalised information based on what they would find helpful, encouraged to share their own thoughts or offered choice in relation to timing or mode of birth. HCPs described uncertainty regarding the evidence around optimal timing of birth as a barrier to involving women in decisions about their care. Training for HCPs in SDM practices, personalised information provision, decision support tools, midwife-led antenatal classes for high-risk women and multiprofessional clinics have been identified as potential facilitators to the adoption of SDM.

A major strength of the study is the recruitment of black, Asian and minority ethnic women to both the research (40%) and in the PPI planning stage, as these women are disproportionately represented in the chronic hypertension in pregnancy population and often under-represented in research. A further strength is the use of multiple methods approaches and analysis underpinned by a theoretical model to improve reliability, validity and generalisability. The structured observations were carried out using a validated tool with high inter-rater reliability.^16^ A limitation of the study is that the case-note review relied on electronic coding of pregnant women with chronic hypertension (or essential hypertension). However, at one hospital, it is likely, based on population trends, that there were additional women with chronic hypertension who had not been coded correctly and, as such, were not identified for case-note review. It is also recognised that the absence of SDM documentation in case notes does not necessarily mean SDM did not occur but rather was not evidenced. It is also acknowledged that each woman only had one third trimester appointment observed, and that further birth planning discussions may have occurred at a different gestation. Nevertheless, the multiple methods integrated analysis, including interviews with women and HCPs, found women often had limited involvement in birth planning, contrary to national guidelines. A final limitation was that data were collected in 2018; however, the 2023 Care Quality Commission Maternity Survey found that in women with pre-existing conditions, experiences of their care either remained the same or worsened over a 5-year period, indicating these results likely remain relevant to current practice.^23 24^

Our study has found that women planning birth who were involved in decision-making felt respected and had more trust in their HCPs. These findings are supported by a recent survey of 14 425 individuals in the USA which found that when clinicians reported they had adopted optimal patient-centred communication practices, patients had significantly increased trust in the clinicians as an information source.^25^ Nevertheless, our study found that neither the principles of SDM set out in the NHS England and Wales NICE 2012 and 2021 SDM guidelines^8 9^ nor the GMC consent guidance (2008) were adopted in full in most cases.^11^ As, through integrated analysis, it was found women were not usually provided personalised information based on what they would find helpful, encouraged to share their own thoughts nor offered choice in relation to timing or mode of birth. These findings concur with recent maternity and neonatal investigations into Shrewsbury and Telford and East Kent maternity NHS Trusts.^26 27^ A recent study of Dutch junior doctors noted professionals adopted a ‘paternalistic’ approach to decision-making rather than an SDM one, as they felt it was their responsibility to ensure the best course of action was achieved.^28^ However, this is in direct opposition to the legal principles of patient autonomy set out in England’s High Court and across Europe.^12^ It is perhaps unsurprising that HCPs in this study had differing views and approaches to decision-making, as in the UK the terms SDM, supported decision-making and informed decision-making, personalised care and informed choice are used interchangeably across different policy documents, with little explanation as to the conceptual models underpinning them and evidence around effective implementation strategies.^29^ In the USA, following a case similar to that of Montgomery, the American Association of Obstetricians and Gynaecologists (2021) published a consensus statement that defines SDM as ‘a model of informed consent which encourages physicians to reframe autonomy as ‘relational’, that is, informed by a patient’s interpersonal relationships and broader social environment. Thus, SDM allows patients to obtain personalised information about their treatment options with the goal of improving their ability to make an autonomous decision’.”^30^ A maternity-based consensus statement on SDM and the conceptual model underpinning it may hasten the move towards SDM.

Analysis of qualitative data in this study has identified evidence-based uncertainty around optimal timing of birth as a barrier to HCPs implementing SDM (with HCPs balancing the possible benefits and harms of earlier and later delivery in women at risk of pre-eclampsia and hypertension-related morbidity). The recently funded WILL Trial aimed to address optimal timing of delivery for women with chronic or gestational hypertension who reach term gestational age and investigate the clinical effectiveness of two timing of delivery approaches but was closed early due to lack of recruitment.^31^ In the absence of research evidence, the NICE Hypertension in Pregnancy Guideline states, ‘for women with chronic hypertension whose blood pressure is lower than 160/110 mm Hg after 37 weeks, with or without antihypertensive treatment, timing of birth and maternal and fetal indications for birth should be agreed between the woman and the senior obstetrician’.^5 6^ However, there is a lack of consensus on what constitutes a maternal and fetal indication for earlier or later term birth. As well as the emerging concern that the number of pregnant women (without hypertension) being recommended early term birth on the basis of population risk factors such as ethnicity is rising^32^; and that there is also evidence that increased early term birth may be causing iatrogenic harm.^33^ While research into optimal timing of birth is important, training in SDM would better enable HCPs to support women making decisions when there is uncertainty regarding the best course of action.

Analysis of qualitative data in this study has identified training for HCPs in SDM practices, personalised information provision and decision support tools as potential facilitators to adopting SDM. All of these are more widely acknowledged as facilitators to implementing SDM into the NHS and other healthcare organisations.^34^ Novel findings in this study include the identification of midwife-led antenatal classes for high-risk women and interdisciplinary antenatal clinics as potential facilitators to the adoption of SDM. There is a paucity of data on the current provision of antenatal classes, but prepandemic, less than 30% of pregnant women were offered NHS-run antenatal classes, with figures now likely lower.^35^ Women also felt more joined up antenatal care where midwives, obstetricians and physicians provided care in interdisciplinary clinics, and where common comorbidities such as hypertension and diabetes are managed in one clinic would reduce decisional conflict and improve SDM. Developing a national consensus statement on maternal SDM and consent, and clarifying the conceptual model underpinning it, is likely to facilitate more widespread adoption of SDM. HCP training in SDM (including how to support women to make decisions about their care when there is uncertainty regarding the best option) would likely improve implementation of SDM in women with chronic hypertension in pregnancy making decisions about birth. Implementing decision support tools and adopting the ‘three talk’ model would also likely improve SDM. Future studies may also wish to investigate the impact of midwifery-led antenatal classes for high-risk women and interdisciplinary antenatal clinics on women’s experiences of birth-related decision-making and their birth outcomes.

## Conclusions

HCPs delivering care to pregnant women with high blood pressure were aligned with most medical communication practices (set out in the Calgary-Cambridge communication guide). Pregnant women with hypertension who described being engaged in SDM about ‘timing’ and ‘mode of birth’ described developing a more trusting relationship with their maternity team and described being satisfied with their care. However, most women were not engaged in SDM when decisions about timing and mode of birth were made. Strategies to promote more widespread adoption of SDM are likely to improve the experiences of women with high blood pressure who are making decisions about childbirth. Future research should investigate the effectiveness of active implementation of SDM on women’s experiences of birth-related decision-making and birth outcomes.

## Supplementary material

10.1136/bmjopen-2024-094607 online supplemental file 1

10.1136/bmjopen-2024-094607 online supplemental file 2

## Data Availability

Data are available on reasonable request.
