# Supplementary material for: What matters to you? Improving the adoption of shared decision-making for birth planning in women with chronic hypertension: a multicentre multiple methods study
Source: BMJ Open. 2025 Jun 17;15(6):e094607. doi: 10.1136/bmjopen-2024-094607 (PMC12182189; doi:10.1136/bmjopen-2024-094607)
Supplement: online supplemental file 1 [file bmjopen-15-6-s001.docx]

**Supplementary File 1**

**Women’s experience of their care (third trimester)**

• Introductions

• Confidentiality

• During this pregnancy you have been treated for chronic hypertension, is that right?

• Can you tell me a bit about your high blood pressure in the last few weeks of your pregnancy?

• How satisfied are you with the results of your care during the last few weeks of pregnancy?

• Thinking about your care during your pregnancy…can you tell me about any information about your choices you were given for maternity care?

• How do you feel about the information given (or not given) to help you decide about your birth?

• How do you feel about he timing of this information to help you decide about your birth?

• What are your feeling regarding confidence and trust in the staff caring for you?

Reference: International Consortium for Health Outcomes Measurement. Pregnancy and Childbirth Standard Set and Reference Guide. 2016. http://www.ichom.org/medical-conditions/pregnancy-and-childbirth/.
