# Supplementary material for: What matters to you? Improving the adoption of shared decision-making for birth planning in women with chronic hypertension: a multicentre multiple methods study
Source: BMJ Open. 2025 Jun 17;15(6):e094607. doi: 10.1136/bmjopen-2024-094607 (PMC12182189; doi:10.1136/bmjopen-2024-094607)
Supplement: online supplemental file 2 [file bmjopen-15-6-s002.docx]

**Supplementary File 2. Observations of eighteen third trimester birth planning consultations, denominator based on the applicability of each statement the observed consultation.**

| ***Calgary Cambridge*** | **% achieved (nominator and denominator)** |
| --- | --- |
| **INITIATING THE SESSION:** |  |
| 1. Greets patient and obtains patient’s name* | 94.4 (17/18) |
| 2. Introduces self, role and nature of interview; obtains consent if necessary | 88.8 (16/18) |
| 3. Demonstrates respect and interest, attends to patient’s physical comfort | 94.4 (17/18) |
| 4. Identifies the patient’s problems or the issues that the patient wishes to address with appropriate opening question (e.g. “What problems brought you to the hospital?” or “What would you like to discuss today?” or “What questions did you hope to get answered today?”) | 56.2 (9/18) |
| 5. Listens attentively to the patient’s opening statement, without interrupting or directing patient’s response | 100 (18/18) |
| 6. Confirms list and screens for further problems (e.g. “so that’s headaches and tiredness; anything else……?”) |  |
| 7. Negotiates agenda taking both patient’s and physician’s needs into account | 29.4 (5/18) |
| **GATHERING INFORMATION** |  |
| 8. Encourages patient to tell the story of the problem(s) from when first started to the present in own words | 83.3 (15/18) |
| 9. Uses open and closed questioning technique, appropriately moving from open to closed | 88.8 (16/18) |
| 10. Listens attentively, allowing patient to complete statements without interruption and leaving space for patient to think before answering | 94.4 (17/18) |
| 11. Facilitates patient's responses verbally and non–verbally e.g. use of encouragement, silence, repetition, paraphrasing, interpretation | 94.4 (17/18) |
| 12. Picks up verbal and non–verbal cues (body language, speech, facial expression, affect); checks out and acknowledges as appropriate | 100 (18/18) |
| 13.Clarifies patient’s statements that are unclear or need amplification (e.g. “Could you explain what you mean by light headed") | 77.8 (17/18) |
| 14. Periodically summarises to verify own understanding of what the patient has said; invites patient to correct interpretation | 94.4 (17/18) |
| 15. Uses concise, easily understood questions and comments, avoids or adequately explains jargon | 100 (18/18) |
| 16. Establishes dates and sequence of events | 83.3 (17/18) |
| 17. Actively determines and appropriately explores patient’s ideas , concerns, expectations and effects on life | 72.2 (13/18) |
| 18. Encourages patient to express feelings | 38.9 (7/18) |
| **PROVIDING STRUCTURE** |  |
| 19. Summarises at the end of a specific line of inquiry to confirm understanding before moving on to the next section | 83.3 (15/18) |
| 20. Progresses from one section to another using signposting, transitional statements; includes rationale for next section | 88.8 (16/18) |
| 21. Structures interview in logical sequence | 88.8 (16/18) |
| 22. Attends to timing and keeping interview on task | 88.8 (16/18) |
| **BUILDING RELATIONSHIP** |  |
| 23. Demonstrates appropriate non–verbal behaviour • eye contact, facial expression • posture, position & movement • vocal cues e.g. rate, volume, tone | 100 (18/18) |
| 24. If reads, writes notes or uses computer, does in a manner that does not interfere with dialogue or rapport | 100 (18/18) |
| 25. Demonstrates appropriate confidence | 100 (18/18) |
| 26. Accepts legitimacy of patient’s views and feelings; is not judgmental | 83.3 (15/18) |
| 27. Uses empathy to communicate understanding and appreciation of the patient’s feelings or predicament; acknowledges patient's views and feeling | 50.0 (9/18) |
| 28. Provides support: expresses concern, understanding, willingness to help; acknowledges coping efforts and offers partnership | 38.9 (7/18) |
| 29. Deals sensitively with embarrassing and disturbing topics and physical pain, including when associated with physical examination** | 100 (7/7) |
| 30. Shares thinking with patient to encourage patient’s involvement (e.g. “What I’m thinking now is....”) | 94.4 (17/18) |
| 31. Explains rationale for questions or parts of physical examination that could appear to be non-sequiturs | 83.3 (17/18) |
| 32. During physical examination, explains process, asks permission | 93.8 (17/18) |
| **EXPLANATION AND PLANNING** |  |
| 33. Chunks and checks: gives information in manageable chunks, checks for understanding, uses patient’s response as a guide to how to proceed | 88.8 16/18 |
| 34. Assesses patient’s starting point: asks for patient’s prior knowledge early on when giving information, discovers extent of patient’s wish for information | 33.3 (6/18) |
| 35. Asks patients what other information would be helpful e.g. aetiology, prognosis | 22.2 (4/18) |
| 36. Gives explanation at appropriate times: avoids giving advice, information or reassurance prematurely | 83.3 (15/18) |
| 37. Organises explanation: divides into discrete sections, develops a logical sequence | 88.8 (16/18) |
| 38. Uses explicit categorisation or signposting | 88.8 (16/18) |
| 39. Uses repetition and summarising to reinforce information | 88.8 (16/18) |
| 40. Uses concise, easily understood language, avoids or explains jargon | 94.4 (17/18) |
| 41. Uses visual methods of conveying information: diagrams, models, written information and instructions | 38.8 (7/18) |
| 42. Checks patient’s understanding of information given (or plans made): e.g. by asking patient to restate in own words; clarifies as necessary | 44.4 (8/18) |
| **Achieving a shared understanding: incorporating the patient’s perspective** |  |
| 43. Relates explanations to patient’s illness framework: to previously elicited ideas, concerns and expectations | 55.6 (10/18) |
| 44. Provides opportunities and encourages patient to contribute: to ask questions, seek clarification or express doubts | 83.3 (15/18) |
| 45. Picks up verbal and non-verbal cues e.g. patient’s need to contribute information or ask questions, information overload, distress | 88.8 (16/18) |
| 46. Elicits patient's beliefs, reactions and feelings re information given, terms used; acknowledges and addresses where necessary | 55.6 (10/18) |
| **Planning: shared decision making** |  |
| 47. Shares own thinking as appropriate: ideas, thought processes, dilemmas | 100 (18/18) |
| 48. Involves patient by making suggestions rather than directives | 61.1 (11/18) |
| 49. Encourages patient to contribute their thoughts: ideas, suggestions and preferences | 55.6 (10/18) |
| 50. Negotiates a mutually acceptable plan | 66.7 (12/18) |
| 51. Offers choices: encourages patient to make choices and decisions to the level that they wish | 27.8 (5/18) |
| 52. Checks with patient if accepts plans, if concerns have been addressed | 66.7 (12/18) |
| **CLOSING THE SESSION** |  |
| 53. Contracts with patient re next steps for patient and physician | 100 (18/18) |
| 54. Safety nets, explaining possible unexpected outcomes, what to do if plan is not working, when and how to seek help | 100 (18/18) |
| 55. Summarises session briefly and clarifies plan of care | 88.8 (16/18) |
| 56. Final check that patient agrees and is comfortable with plan and asks if any corrections, questions or other items to discuss | 83.3 (15/18) |

***The term patient (as set out in the Calgary Cambridge Guide) refers in this case to pregnant women and birthing people**

****This was only applicable to seven women who were undergoing a membrane sweep in antenatal clinic**
