# Supplementary material for: What matters to you? Improving the adoption of shared decision-making for birth planning in women with chronic hypertension: a multicentre multiple methods study
Source: BMJ Open. 2025 Jun 17;15(6):e094607. doi: 10.1136/bmjopen-2024-094607 (PMC12182189; doi:10.1136/bmjopen-2024-094607)
Supplement: Supplementary file 3 [file bmjopen-2024-094607.reviewer_comments.pdf]

PEER REVIEW HISTORY

BMJ Open publishes all reviews undertaken for accepted manuscripts. Reviewers are asked to complete a checklist review form and are provided with free text boxes to elaborate on their assessment. These free text comments are reproduced below.

ARTICLE DETAILS

Title (Provisional)

What matters to you? Improving the adoption of shared decision-making for birth planning in women with chronic hypertension.

Authors

Whybrow, rebecca; Chappell, Dr Lucy; Webster, Louise; Girling, Joanna; Brown, Heather; Wilson, Hannah; Green, Marcus; Sandall, Jane

---

VERSION 1 - REVIEW

**Reviewer**	**1**
**Name**	**McKnight , Christine**
**Affiliation**	**Cardiff Metropolitan University, Cardiff School of Health & Sport Sciences**
**Date**	**16-Dec-2024**
**COI**	**None**

This is an important area for research

Comments:

Introduction: The NICE guidance referred to in the introduction is out of date and is now updated to NG133 which was last reviewed in 2023. The guidance that authors refer to GC107 was published in 2010. It is critical that authors use the most up to date evidence and guidance in their rationale for studies. Other studies within the introduction are also what would be considered older although studies such as Elwyn et al 2012 would be considered appropriate as it is a seminal article on shared decision making. The shared decision making guidelines referred to in reference 7 (NICE CG138) are also not the most up to date guidelines which is NICE CG197. Although the authors do set out a rationale for the research it is problematic that they have used old NICE guidance.Where is the research aim/question? Would expect to see it either end of introduction or beginning of methods - it is not clearly stated.

Methods: There is confusion about how the qualitative research element was analysed. Firstly, the authors say that the research is informed by the the Three Talk Model but then go

on to say that data was inductively analysed. There is no mention of materials such as participant information sheets etc.

Results:Descritptive statistics set out clearly although some of the interpretation is quite short. Qualitative results are not clearly introduced. They are set out utilising a framework (Three Talk Model) this is problematic as that is not fully set out in the methods section which states, several times, that results were inductively analysed. The interpretation of the qualitative data is very short which could be due to word limitation. Maybe the authors have tried to report too much in one article?

Discussion:Again, at times the results are discussed in relations to guidelines that are out of date.The strengths of the study are clearly set out especially with regard to the inclusion of participants who are underrepresented. Although mentions on page 4 of the document limitations are not set out in the discussion as the strengths are.

General comments: Check grammar and also use of contractions.

**Reviewer**	**2**
**Name**	**Suhaimi, Julia**
**Affiliation**	**University of Malaya, Primary Care Medicine**
**Date**	**12-Mar-2025**
**COI**	**None**

Thank you for the manuscript. The findings from this research are indeed useful to improve maternal health in your healthcare centres.

However, I would like to clarify a few statements from your manuscript.

(Page 7, lines 21-23): "At hospital Trust 1 all women who had given birth over the final 3 months of 2017 were included."

What do the authors mean by "all"? Is it all women with chronic hypertension OR all women (irrespective of underlying medical conditions)?

(page 8, lines 3-7): It was stated that 18 of the 3rd trimester antenatal appointments between 18 pregnant women with chronic hypertension and their healthcare provider (doctors/midwives) were observed by a researcher.

How did the researcher observe the consultations? Was she in the same consultation rooms as the patient and the doctor/midwife? It is unclear how the observations were made.

The reason I am enquiring about this is because, in the results, it was reported that most of the HCPs were aligned with the Calgary-Cambridge communication guide. Were they self-conscious that they were being observed hence the positive observation?

Table 1: It is surprising to see that the majority of pregnant women with chronic hypertension were younger than 35 years old. Is this a common finding in the UK? It is known that advanced age (usually more than 35 years old) is a risk factor for developing hypertension in pregnancy.

It is clear from your finding that pregnant women appreciate an early discussion on timing and mode of delivery as opposed to the HCPs who prefer the have the discussion in the 3rd trimester once more clinical information are made available.

Indeed the HCPs' training in SDM would likely encourage more SDM in caring for pregnant women with chronic hypertension.

VERSION 1 - AUTHOR RESPONSE

The NICE guidance referred to in the introduction is out of date and is now updated to NG133 which was last reviewed in 2023. The guidance that authors refer to GC107 was published in 2010. It is critical that authors use the most up to date evidence and guidance in their rationale for studies.<br><br>Other studies within the introduction are also what would be considered older although studies such as Elwyn et al 2012 would be considered appropriate as it is a seminal article on shared decision making.<br><br>The shared decision making guidelines referred to in reference 7 (NICE CG138) are also not the most up to date guidelines which is NICE CG197. Although the authors do set out a rationale for the research it is problematic that they have used old NICE guidance.	The authors intended to explain the standards at the time of data collection but acknowledge this could be confusing. The recommendation remained unchanged in 2019 and therefore both the 2010 and 2019 ref have now been included.<br><br>This has been left as indicated by the reviewer.<br><br>As above ,it is understood this reference may cause confusion. We have added the most recent NICE SDM reference CG197 to this reference as the definitions and recommendations remain unchanged.
Where is the research aim/question? Would expect to see it either end of introduction or beginning of methods - it is not clearly stated.	The study aims and objectives have been more clearly set out at the start of the methods section as follows:<br><br>Line 114 -120<br><br>Informed by the Three Talk Model,[14] the study aims to: Explore the role of shared decision-making in the implementation

	of evidence-based practice in women with chronic hypertension. Objectives: 1. describe the care being implemented for women with chronic hypertension in pregnancy across England; 2. investigate the current uptake of evidence-based practice and explore the determinants associated with their implementation for women with chronic hypertension in pregnancy; 3. investigate the barriers and facilitators in the provision of antenatal care for women with chronic hypertension in pregnancy.
Methods: There is confusion about how the qualitative research element was analysed. Firstly, the authors say that the research is informed by the Three Talk Model but then go on to say that data was inductively analysed.	This has been clarified on Line 202 as follows:<br><br>The interviews were audio transcribed, coded and thematically analysed using a deductive approach[17] informed by The Three Talk Model for SDM.
There is no mention of materials such as participant information sheets etc.	Line 199 has been updated as follows: The interviews were carried out by a midwife researcher (RW) following informed consent whereby participants were provided with information about the study and given time to ask questions prior to providing consent and took place in privacy away from the clinical setting.
Results:<br>Descritptive statistics set out clearly although some of the interpretation is quite short.	In alignment with reviewer 2's comment and this comment we have updated the descriptive statistics as follows:<br><br>Line 248:<br><br>Approximately two-fifths of those recruited to the study were of Black, Asian and minority ethnic backgrounds, approximately one-third had a BMI over 30 kg/m2 and approximately one-third were over the age of 35. These demographics are aligned with Webster and colleagues' UK CHT cohort study published in 2019.[21]
Qualitative results are not clearly introduced. They are set out utilising a framework (Three Talk Model) this is problematic as that is not fully set out in	The authors have sought to strike a balance between manuscript length and integration of multiple methods data (which strengthens the study's findings).

the methods section which states, several times, that results were inductively analysed. The interpretation of the qualitative data is very short which could be due to word limitation. Maybe the authors have tried to report too much in one article?	We have clarified on Line 197 that the interviews were analysed deductively.<br><br>The interviews were audio transcribed, coded and thematically analysed using a deductive approach[17] informed by The Three Talk Model for SDM.
Discussion: Again, at times the results are discussed in relations to guidelines that are out of date.	In line with the background, we have updated the NICE SDM reference and the NICE hypertension in pregnancy reference to include both the reference at time of data collection and current unchanged guidance.
The strengths of the study are clearly set out especially with regard to the inclusion of participants who are underrepresented. Although mentions on page 4 of the document limitations are not set out in the discussion as the strengths are	The following methodological limitations are set out in both the limitations section preceding the full text and within the discussion. We have clarified in the discussion section that these are limitations.<br><br>A limitation of the study is that the case-notes review relied on electronic coding of pregnant women with chronic hypertensions (or essential hypertension) however at one hospital it is likely, based on population trends, that there were additional women with chronic hypertension who had not been coded correctly; and as such were not identified for case-note review.<br><br>A final limitation was that data were collected in 2018; however, the 2023 Care Quality Commission Maternity Survey found that in women with pre-existing conditions, experiences of their care either remained the same or worsened over a five year period, indicating these results likely remain relevant to current practice.[23, 24]
General comments: Check grammar and also use of contractions.	Thank you. We have reviewed the grammar and contraction across the manuscript.
(Page 7, lines 21-23): "At hospital Trust 1 all women who had given birth over the final 3 months of 2017 were included."	Line 164 has been changed to:

What do the authors mean by "all"? Is it all women with chronic hypertension OR all women (irrespective of underlying medical conditions)?	At hospital Trust 1 all women with CHT who had given birth over the final 3 months of 2017 were included
(page 8, lines 3-7): It was stated that 18 of the 3rd trimester antenatal appointments between 18 pregnant women with chronic hypertension and their healthcare provider (doctors/midwives) were observed by a researcher.<br>How did the researcher observe the consultations? Was she in the same consultation rooms as the patient and the doctor/midwife? It is unclear how the observations were made.<br>The reason I am enquiring about this is because, in the results, it was reported that most of the HCPs were aligned with the Calgary-Cambridge communication guide. Were they self-conscious that they were being observed hence the positive observation?	Line 189 has been updated as follows: The midwife researcher carried out the observations in person in the antenatal clinic room. During observations, data about birth planning were recorded using the Calgary-Cambridge communication guide[16] chosen for validity in relation to the research question, and its high inter-rater reliability. Professionals were told antenatal care was being observed but not specifically using the Calgary-Cambridge communication guide.
Table 1: It is surprising to see that the majority of pregnant women with chronic hypertension were younger than 35 years old. Is this a common finding in the UK? It is known that advanced age (usually more than 35 years old) is a risk factor for developing hypertension in pregnancy.	Line 251 has been updated as follows: Approximately two-fifths of those recruited to the study were of Black, Asian and minority ethnic backgrounds, approximately one-third had a BMI over 30 kg/m2 and approximately one-third were over the age of 35. These demographics are aligned with Webster and colleagues' UK CHT cohort study published in 2019.[21]
It is clear from your finding that pregnant women appreciate an early discussion on timing and mode of delivery as opposed to the HCPs who prefer the have the discussion in the 3rd trimester once more clinical information are made available.<br><br>Indeed the HCPs' training in SDM would likely encourage more SDM in caring for pregnant women with chronic hypertension.	Thank you for your review of the manuscript we are pleased these findings are helpful to HCPs discussing timing of birth with women who have CHT. Future work will explore how SDM training for HCPs can be improved.

VERSION 2 - REVIEW

Reviewer 1

Name McKnight , Christine

Affiliation Cardiff Metropolitan University, Cardiff School of Health & Sport Sciences

Date 12-May-2025

COI

I have previously reviewed this paper and have now examined the revisions that were suggested previously. I find that all the revisions requested have been adequately dealt with in the updated revised manuscript. Thank you for taking the time and effort to address these.
